# *Trypanosoma cruzi*–Host Cell Interaction

**DOI:** 10.3389/fimmu.2014.00339

**Published:** 2014-08-04

**Authors:** Wanderley De Souza

**Affiliations:** ^1^Laboratório de Ultraestrutura Celular Hertha Meyer, Centro de Ciências da Saúde, Instituto de Biofísica Carlos Chagas Filho, Universidade Federal do Rio de Janeiro, Rio de Janeiro, Brazil; ^2^INMETRO – Instituto Nacional de Metrologia, Qualidade e Tecnologia, Rio de Janeiro, Brazil

**Keywords:** parasitic protozoa, parasite-host cell interaction, cell-to-cell recognition, *Trypanosoma cruzi*, Chagas Disease

Chagas disease was discovered by Carlos Chagas in Brazil in 1909 (1). It is caused by the pathogenic protozoan *Trypanosoma cruzi*, member of Trypanosomatidae family, Kinetoplastida order. Chagas disease is recognized by the World Health Organization as one of the main neglected tropical diseases, affecting about 8–15 million individuals in 18 countries in Central and South America, where it is endemic, as well as countries in North America and Europe (2). At least 30 million people are at risk. Public health programs have significantly reduced transmission of Chagas disease, however, blood and organ transplant transmission in non-endemic countries and a growing number of food borne (especially fruit juices) infections still require special attention. In addition, an increase in the rate of infection in the Amazon region has become a challenge for the control of Chagas disease (3).

*Trypanosoma cruzi* presents a complex life cycle both in the vertebrate and invertebrate hosts, involving dramatic changes in cell shape (4). Its life cycle involves several developmental stages that are known as amastigotes, epimastigotes, and trypomastigotes. The first two stages are able to divide inside and outside host cells, respectively. The trypomastigote and amastigote stages are also able to infect host cells where they multiply, amplifying the number of parasites, and releasing millions of the infective trypomastigote forms in the intercellular spaces.

This thematic issue deals with the ability of *T. cruzi* to penetrate into host cells. In the first article, de Souza and de Carvalho (5) make a review of the concept of active penetration and suggest that *T. cruzi* always penetrates the host cell through an endocytic process with the formation of a transient parasitophorous vacuole. The second article by Barrias et al. (6) reviews the various mechanisms of endocytosis, which are used by the protozoan to gain access to the intracellular portion of the host cells. These include processes such as classical phagocytosis, participation of membrane rafts, macropinocytosis, and clathrin-mediated endocytosis. In the third article, Calvet et al. (7) analyze in detail the process of interaction of *T. cruzi* with cardiomyocytes, an important host cell, because *in vivo* many of the parasite strains have a tropism for the heart. In the fourth article, Tonelli et al. (8) call the attention to the fact that most probably a large number of molecules are involved in the process of protozoan–host cell interaction and discuss the use of more powerful technologies, such as peptide-based phage display and oligonucleotide-based aptamer techniques. Using these approaches, the results obtained by the group highlight the importance of members of the 85-kDa family on the process of interaction. In the fifth article, Freire-de-Lima et al. (9) point out the relevance of a unique system of sialoglycoproteins and sialyl-binding proteins, which in the case of *T. cruzi* are represented by trans-sialidases. These proteins are involved in parasite–host cell recognition, infectivity, and survival. The sixth and seventh articles by Nde et al. (10) and Ferreira et al. (11) respectively, analyze the role played by components of the extracellular matrix during the interaction of the trypomastigote and amastigote forms of *T. cruzi* with the host cells. Infective trypomastigotes up-regulate the expression of laminin-gamma^−1^ and thrombospondin to facilitate recruitment of parasites to enhance cell infection. The extracellular matrix interactome network seems to be regulated by *T. cruzi* and its gp 83 ligand. The eighth article by Maeda et al. (12) reviews the cell signaling process that takes place during the interaction of metacyclic trypomastigotes, infective forms found in the invertebrate host, with host cells. Special emphasis is given to intracellular calcium mobilization and the triggering the exocytosis of host cell lysosomes during the interaction process mediated by a surface-expressed parasite glycoprotein of 82 kDa. This process leads to the activation of mammalian target of rapamycin (mTor), phosphatidylinositol 3-kinase, and protein kinase C. The last article, by Scharfstein et al. (13) initially analyses the process by which *T. cruzi* trypomastigotes elicit an inflammatory edema that stimulates protective type-1 effector cells through the activation of the kallikrein–kinin system, providing a framework to investigate the intertwined proteolytic circuits that couple the anti-parasite immunity to inflammation and fibrosis.

## Conflict of Interest Statement

The author declares that the research was conducted in the absence of any commercial or financial relationships that could be construed as a potential conflict of interest.
